# A 3-year review of MRI safety incidents within a UK independent sector provider of diagnostic services

**DOI:** 10.1259/bjro.20180006

**Published:** 2019-04-30

**Authors:** Darren Hudson, Andrew P Jones

**Affiliations:** 1 InHealth Group, Buckinghamshire, UK; 2 Christie Medical Physics and Engineering, The Christie NHS Trust, Manchester, UK

## Abstract

A review of MRI safety incidents conducted over a 3-year period for a large independent sector diagnostic imaging provider in the UK. The review took a systematic approach using reports logged on an internal incident reporting system that were then categorised and analysed for themes and trends. Notable cases and actions taken are also described from within the period. MRI safety-related events made up 7.5% of the total number of incident reports submitted and 15.5% of all MRI-related reports. The MR safety-related incidence report rate was 0.05% (1 per 1987 patients), which is relatively low considering the number of patients seen in our facilities each day. Internal MRI safety events indicated the main trends to be around referral of contraindicated devices (32% of reports) and failure in the screening process (21.5%—either due to unexpected implants or being unable to confirm safety). To improve practice and work to reduce incidents, advice and instructional materials were developed. The review suggests a potential approach to categorisation of MRI-related safety events which could allow comparisons to be made across organisations, helping to look for trends and guide learning. It also provides insight into the state of MRI safety within the organisation, a rationale for some of the interventions introduced to improve safety, and discussion around common issues arising in MRI safety.

## Introduction

InHealth is an independent sector provider of diagnostic and healthcare solutions within the UK. Approximately, half of the business activity is through the provision of MRI services. Various models of service delivery operate; static, semi-static and mobile, either hospital-based or in the community, and predominantly for the National Health Service although also for some private healthcare providers. The organisation operates over 70 scanners across the UK, including 3 T systems and one 1.2 T open system, from a range of manufacturers. Almost half a million patients are scanned each year across the organisation.

A review of MRI-related safety incidents has been carried across the wide-reaching MRI services following internal governance structure and processes. The review provides an overview of how incidents relating to MRI safety are broken down for more meaningful analysis and how some of this analysis compares with the literature. An outline of some notable cases and actions taken in response to the trends seen has also been collated. It is hoped that the review will serve to provide others with potential data for benchmarking and continue the discussion around the ever-evolving field of MRI safety. MRI is often considered a safe imaging modality considering the absence of ionising radiation used to acquire images.^1,2^ However, this is only the case when due diligence and correct procedures are applied in order to manage patients safely.

Risks in MRI can be usefully broken down into either direct effects or indirect effects.^3^ Direct effects arise from the electromagnetic fields (EMFs) associated with the main equipment components of the MRI scanner, namely, (a) the static magnetic field (B_0_) which has the potential for inducing transient sensory effects, (b) the applied radiofrequency (RF) pulses which produce a heating effect within body tissues, and (c) the time-varying gradients (dB/dt) which may cause peripheral nerve stimulation.^4^ Indirect effects are secondary to the direct hazards and as a consequence of implications of working practices. Examples of indirect effects are forces exerted on ferromagnetic objects by the main magnetic field, the high acoustic sound levels within the bore of the magnet arising from the forces created by the switched magnetic field gradients, effects on biomedical implants and patient burns from inappropriate use of equipment (*e.g.* electrodes, leads etc.). Direct effects are, in principle, avoided or controlled and risks minimised by the regulated design and operation of the equipment. Indirect effects are controlled and minimised by the application of effective safe working practices. It is, therefore, the management of these indirect hazards and reducing their risks where human factors and the correct compliance with safe working practices are essential to maintain both patient and staff safety.

Direct harm to staff or patients from MRI is low,^5^ and most often when it does occur, it is the result of a series of failings caused by some process failure or human error which are ultimately preventable. Therefore, the logging and investigation of near misses or incidents (collectively termed safety events for the purpose of this review) is key so that lessons can be learned and shared to prevent serious harm from occurring and promote a culture of safety.^6^ This monitoring of trends together with a cycle of continuous learning and improvement is essential for the assurance and delivery of safe effective care in a culture without blame.^1,7^

In terms of further risks associated with the MR system and the MR examination, any complete analysis must include the potential risks associated with gadolinium-based contrast agent (GBCA) administration. Whilst a detailed discussion of the current accepted risks and guidelines around administration are outside of the scope of this review, the potential for contrast-related reactions or extravasations should be included within any recording of incidents related to MRI.

Additionally, the requirement for liquid helium within superconducting magnets may present a hazard in the event of a magnet quench. A blocked or faulty quench pipe system can result in gaseous helium being released during a quench and being vented into the magnet room. In such circumstances, the resulting displacement of oxygen in the magnet room can lead to a potential asphyxiation and cold contact burn risks.

### Categories of reported incidents and data analysis

There is little guidance within the literature on the breakdown and classification of MRI safety events. Much is reported around contrast agent-related incidents but otherwise studies look at varying aspects of MRI-related incidents—including incorrect right/left referrals, scheduling issues, claustrophobia, technical artefacts, failure to scan, and unexpected foreign bodies.^8,9^ The MHRA Guidance document^10^ provides some suggested incidents to report which are more MRI safety specific and include projectiles, RF burns, quench, device malfunction, acoustic noise etc.

The proposed 15 MRI-related subcategories used in this review were compiled from the advice and experiences within the literature and designed to summarise the key causes of MRI safety events that could then be monitored and used for learning. In addition, contrast-related events were divided into reactions, extravasations, and other medicines-related issues. These additional subcategories relating to possible contrast administration incidents were added to the list, producing a total of 18 possible incident subcategories. The subcategories are listed in Table 1.

**Table 1.** MR incident subcategories used in the review

**1**	Projectile	** 7**	Unable to confirm safety	**13**	Faulty oxygen monitor
**2**	Equipment labelling	** 8**	Implant scanned outside of policy	**14**	Quench
**3**	Unauthorised access	** 9**	Unexpected implant or foreign body	**15**	Other MRI-related safety issue
**4**	Burn	**10**	Implant/device related issue	**16**	Adverse contrast reaction
**5**	Peripheral nerve stimulation	**11**	Contraindicated referral	**17**	Extravasation
**6**	Noise complaint	**12**	Faulty/damaged coil	**18**	Other drug-related issue

Activity data from across the organisation were analysed internally from *Kimera software*, the organisations main reporting suite including the data warehouse; (a SAP product called Business Objects Web Intelligence designed to meet *ad* *hoc* reporting and analysis of data), with safety- and drug-related event reports collated via the internal reporting system, *Sentinel* (incident report management system by Vantage Technologies Ltd, UK).

Reports of recorded MRI safety events were generated by filtering fields on the Sentinel system to pick out “MRI”, “safety”, and “contrast” between January 2015 and December 2017 inclusive. Each report was then manually reviewed and subcategorised to better assess any significant trends. Because this is, in most situations, a manual process and due to the increasing volumes recorded, it is a time intensive exercise.^7^ Longer term, use of these subcategories would be better built into the reporting system for categorising at the time of submission, thereby making analysis more automated which will make it less time consuming and manual. Although one of the main difficulties when subcategorising some of the safety events is that in most cases they are multifactorial and associated with other causations,^9^ they may therefore apply to more than one category. Review of each report is therefore still necessary and allows opportunity to better understand what events are occurring. The root cause or the most dominant category was chosen to identify the key aspect leading to the occurrence of an event, but this is an acknowledged limitation to this review and based on the reviewer’s subjective opinion (MRI Clinical Lead for the organisation, qualified for 18 years and MR Safety Certified^™^ by the American Board of MR Safety).

## Results

The 18 subcategories described in Table 1 were used to categorise the events from the Sentinel incident report management system. Table 2 provides a brief description of the nature of each of the subcategories along with examples of the some of the recorded incidents from the Sentinel data.

**Table 2.** MR-related subcategories used for incident recording and a description of the nature of each category

Subcategory	**Definition**	**Example(s**)
***Projectile***	Actual or near miss event where a confirmed, or potentially, ferromagnetic item is taken into the MR Environment.	Nearly all cases involved patients with items found remaining on their person, in pockets.Most commonly coins, keys and mobile phones… in some cases pen knives and in one case a wrench! Patients were asked to remove items and empty pockets but somehow the message wasn’t received or understood.Minor harm, cuts/bruises, did occur in a couple of cases due to the impact of the small projectile.There was a shaver hidden within bed clothes.The most significant near miss was a nurse bringing an O_2_ cylinder into the scan room door—see the “Notable cases” section.
***Equipment labelling***	Ancillary equipment within MR Controlled Access area not labelled, or inadvertently taken into scan room due to lack of labelling/awareness.	Various pieces of equipment identified.The main issues were around non-MRI chairs being taken into scan rooms.
***Unauthorised access***	Access to the MR Controlled Access area by unauthorised staff or members of the public.	Cases noted where members of public entered controlled access area due to issue with door locks or maintenance works.
***Burn***	Patient burn as a result of conduction loop or proximity to transmit body coil etc	Few cases of heating and actual burns.2 tattoo heating cases within the area being scanned3 conduction loop burns—one thumb-thigh and two between thighs.One case related to fibres in clothing (jumper).One case relating to coil cable heating—see the “Notable cases” section.
***PNS***	Painful PNS experienced by a patient.	None noted.
***Noise complaint***	Patient raises concern over noise levels experienced and subsequent temporary hearing irritation.	Generally around lack of awareness and explanation about correct use.
***Unable to confirm safety***	Lack of implant info or unaware of implant *in situ* before attendance, not highlighted by referrer or patient, leading to a delay in the patient pathway/appointment.	Lack of staff awareness around safety policy and correct management of devices/implants.Lack of patient capacity to be able to provide reliable history therefore Medical sign off needed.Limited history of device from referrer or patient.Error and triage/booking and device detected on the dayIssues over devices < 6 weeks post implantation.
***Implant scanned outside of policy***	Device *in situ* generally not scanned under safety policy, but scanned in error, or due to urgency and risk-benefit. May or may not have been discussed with MRSE and appropriate pathway for scanning agreed.	Transpired a patient was scanned with a Conditional PM *in situ* without pathway in place and not being switched to MR mode for scanning—see the “Notable cases” section.
***Unexpected implant or foreign body***	Artefact arising from unexpected metal seen on scans—possibly implant or foreign body *in situ*.	Most common one was unexpected IOFB.Others were unexpected implants from surgery not revealed at screening.Two significant cases of aneurysm clips detected until seen on scan—see the “Notable cases” section.
***Implant/device-related Issue***	Implant scanned and as a direct result of performing MRI is damaged, stops working or causes patient pain or discomfort.Implant or device is taken into scan room but detected before scanning or causing a problem.	Most cases of damage were to hearing aids left in during scanning.One significant incident was around scanning a programmable hydrocephalus shunt which was altered by the magnetic field.Two cases of discomfort in region of prior surgery and clips.
***Contraindicated referral***	Referral made with unsafe implant/device *in situ* causing scan to be cancelled.	Nearly all pacemaker referrals to sites not set up to scan conditional pacemakers.
***Faulty/damaged coils***	Receive coils used with exposed cable, cracks or other visible damage.	None noted—all recorded via fault reporting system.
***Faulty oxygen monitor***	Scan room oxygen monitor alarming or broken, requiring scanning without one.	None noted—all recorded via fault reporting system
***Quench***	Release of helium gas for demagnetisation of scanner.	Two occurrences on mobile trailers pre-commissioning into service.
***Other safety issues***	Any other MR safety-related issue not covered.	Various events, most common ones were around pregnancy—two discovered on scan, three discovered they were pregnant after having said no and had a scan.
***Contrast reaction***	Any suspected or actual reaction following administration of contrast media.	Contrast reactions: all mild-moderate—urticaria, warm, nausea etc. No severe anaphylaxis reported.
***Extravasation***	Any extravasation noted during insertion and use of a peripheral venous catheter.	Predominantly all related to remote pump injections.
***Other drug issues***	Any other issues relating to drugs and medicines management within MRI.	around out of date supplies, and incorrect preparation or administration of contrast.

GP, general practitioner; IOFB, intraocular foreign bodies; PNS, peripheral nerve stimulation.

Examples of recorded incidents are shown to further explain the nature recorded use of the categories.

During the review period, just over 1.3 million patients were scanned across the business with a total number of 4343 MRI-related reports submitted (full breakdown in Table 3a b). This total is made up of 72% (3127) incidents related to static sites and 27% (1173) from mobile units (with 1% relating to head office or call centre reports), arising from a total of 70 MR systems with an approximate 50:50 split between static and mobile-based units. These incident reports accounted for 44% of all events documented which was expected, as this approximately equates to the same percentage of activity that MRI makes up within the business (the remainder being other modalities), and the reporting rate overall for MRI was 0.33% (1 in 308 patients). MRI safety-related events made up 7.5% of the total number of incidence reports submitted and 15.5% of all MRI-related reports (674 total). The MR safety-related incidence report rate was 0.05% (1 per 1987 patients).

**Table 3.** (a) shows the breakdown of all incidents reported within the business between January 2015 and December 2017, highlighting MRI related, MRI safety and MR contrast related data (b) shows the total number of patients scanned during this period and the corresponding incident rates for all incidents, MRI incidents and MRI safety incidents

(a)					
Period	Total no of incidents (b)	Total no of MRI incidents (c)	Total no of MR Safety	Total no of MR contrast	Total no of MR safety + contrast (d)
Jan–Jun 2015	938	357	53	30	83
Jul–Dec 2015	938	358	43	30	73
Jan–Jun 2016	2399	1276	62	38	100
Jul–Dec 2016	1839	919	101	37	138
Jan–Jun 2017	1639	740	114	35	149
Jul–Dec 2017	1654	693	92	39	131
	9407	4343	465	209	674

(b)					
Period	Total no of MRI Patients (a)	Incident report % for MRI = c/b*100	Incident report rate for MRI = c/a*100	Incident report % for MR Safety = d/b*100	Incident report rate for MR Safety = d/a*100
Jan–Jun 2015	210,972	38%	0.169	8.85%	0.039
Jul–Dec 2015	216,323	38%	0.165	7.78%	0.034
Jan–Jun 2016	217,778	53%	0.586	4.16%	0.046
Jul–2016	211,544	50%	0.434	7.50%	0.065
Jan–Jun 2017	217,860	45%	0.34	9.10%	0.068
Jul–Dec 2017	265,025	42%	0.26	7.92%	0.049
	1,339,502	**44%**	**0.3257**	**7.55%**	**0.05017**

The recorded data (outlined in Table 4) shows how reporting within MRI has increased from 2015 to 2017, with a significant rise in the first half of 2016. This period happened to coincide both with an internal drive on reporting incidents and near misses across the organisation, and use of Sentinel for logging and managing failures to scan, both of which were predominantly not safety related. Reports since remain almost double that in 2015 demonstrating increased awareness and promotion of a wider culture of reporting. Specific MRI safety-related events remain generally constant throughout at 7–9% (except the early 2016 period which was reduced due to the increase in other reported events as described).

**Table 4.** Breakdown of incidents by subcategory for 6 monthly periods between 2015 and 2017

Sub category	**Event reports per collection period**						**No of event reports**	**Incidence rate**	
	**Jan–Jun 15**	**Jul–Dec 15**	**Jan–Jun 16**	**Jul–Dec 16**	**Jan–Jun 17**	**Jul–Dec 17**		**% per exam**(**1,339,502**)	**% per event report**
Projectile	**5**	**3**	**4**	**4**	**10**	**6**	32	0.0024	4.75
Equipment labelling	**2**	**1**	**1**	**0**	**1**	**0**	5	0.00037	0.74
Unauthorised access	**0**	**1**	**0**	**0**	**1**	**2**	4	0.00030	0.6
Burn	**2**	**0**	**2**	**2**	**2**	**2**	10	0.00075	1.48
PNS	**0**	**0**	**0**	**0**	**0**	**0**	0	0	0
Noise complaint	**0**	**2**	**2**	**2**	**0**	**0**	6	0.00045	0.89
Unable to confirm safety	**14**	**5**	**7**	**20**	**12**	**19**	77	0.00570	11.4
Implant scanned outside of policy	**1**	**0**	**0**	**1**	**0**	**0**	2	0.00015	0.3
Unexpected implant or foreign body	**4**	**10**	**10**	**15**	**16**	**13**	68	0.0051	10.1
Implant/device-related Issue	**0**	**4**	**5**	**7**	**4**	**0**	20	0.0015	2.97
Contraindicated referral	**21**	**15**	**28**	**45**	**62**	**45**	216	0.016	32
Faulty/damaged coils	**0**	**0**	**0**	**0**	**0**	**0**	0	0	0
Faulty oxygen monitor	**0**	**0**	**0**	**0**	**0**	**0**	0	0	0
Quench	**0**	**0**	**0**	**2**	**0**	**0**	2	0.00015	0.3
Other	**4**	**2**	**3**	**3**	**6**	**5**	23	0.0017	3.41
Adverse contrast reaction	**20**	**14**	**29**	**21**	**22**	**18**	124	0.0093	18.4
Extravasation	**5**	**12**	**5**	**7**	**7**	**17**	53	0.0040	7.86
Other drug-related Issue	**5**	**4**	**4**	**9**	**6**	**4**	32	0.0024	4.75
**Overall totals**	**83**	**73**	**100**	**138**	**149**	**131**	674	0.05	100

PNS, peripheral nerve stimulation.

The bold rows relate to the top ranking incidents as discussed in the paper.

**Figure 1.**
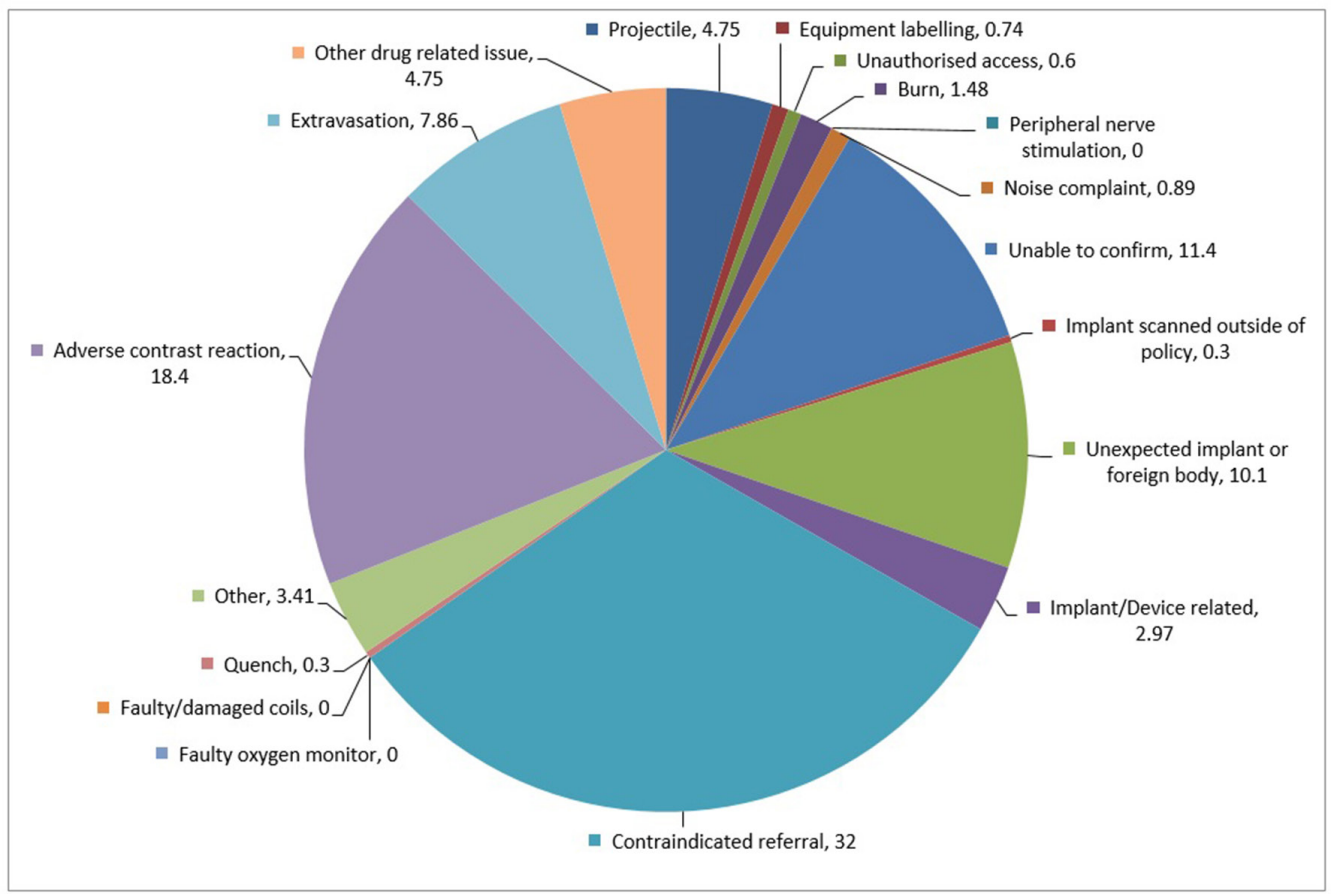
Pie chart of incident subcategory frequency (as percentage of MR safety-related incidents reported).

The breakdown of the subcategories in Figure 1 highlights the largest category of MR safety reports were for contraindicated referrals which made up 32% of all MR safety reports, at a rate of 0.016 (1 per 6201 patients). This was followed by 18.4% being characterised as adverse contrast reactions, 11.4% due to an inability to confirm patient safety prior to scan, and 10.1% from unexpected implants or foreign bodies.

Events relating to indirect effects associated with MRI were low; projectiles accounted for 4.75% of reports, 1.48% were burns (or heating-related issues), and 0.89% were noise-related complaints. No incidence of “reportable painful” peripheral nerve stimulation was noted as per the category definitions (Table 2). There were no equipment-related faults (although these are generally logged on separate fault reporting systems and unless a potential clinical risk is identified, they are not logged on Sentinel).

Of the drug-related incidents occurring in MRI; 60% were reactions and 25% were extravasations related to the use of contrast agents which are not seen as frequently in MRI compared with CT, where a pressure injector is used more often.^8^ The remaining 15% related to other issues such as cardiac drugs, medicines management, and dynamic scan timing. One limitation of the review was that an adverse drug rate could not be calculated due to data on the number of contrast examinations performed being inaccurate as a result of incorrect exam coding and variation in information systems used across different areas of the business.

### Notable cases

As part of the incident management process for the organisation, all reports are reviewed by the Clinical Quality team weekly and any concerning trends or significant incidents trigger a “Rapid Alert” from the MRI Clinical Lead to all staff. The Rapid Alert system is used as a method for feeding back to all MRI staff on issues of urgent importance. The system uses electronic communications alert that all staff are trained to respond to with high priority. Other trends are fed back to staff monthly via a newsletter in order to keep them informed of any shared learning opportunities. It would be good to see more MRI specific sharing of reported events across MRI teams to raise awareness and share lessons learned.

**Figure 2.**
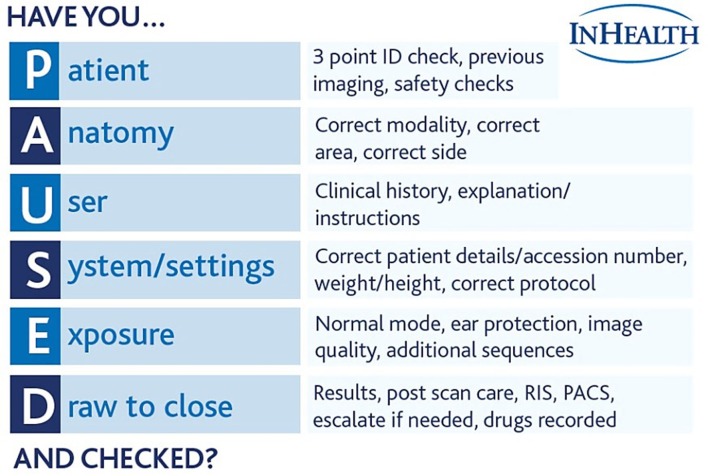
“Have you PAUSED and checked?” checklist passed out to all staff based on the Society of Radiographer’s campaign material.

Over the review period, five alert notifications were issued as a result of potentially serious incidents and procedures that came to light due to reported safety events. Table 5 summarises the alerts, describing the assessed root cause and the resulting outcome and action. Alerts 1 and 2 represent failures around responsibility of referrers and effective communication. Whilst referrers do have responsibility for identifying any MR unsafe implants or other contraindications for their patient,^10^ the lack of information or a failure to source the accurate information for patients on the MR request card is a frequent occurrence. The problems with effective communication within the MRI team recognises the challenges of having multidisciplinary staff with varying levels of knowledge and experience relating to safety. The senior MR radiographer as the MR Supervisor ultimately has the final responsibility for patient safety on a daily basis. However, the shared roles within busy departments can lead to weaknesses in the patient screening process. The alert provided the opportunity to introduce an adapted “Have you PAUSED and checked” based on the Society of Radiographer’s campaign to act as aide memoire for staff when dealing with patients to ensure all suitable checks are carried out throughout the examination. Documentation for “Have you PAUSED and checked” (Figure 2) was passed out to all staff by means of a lanyard tag for ease of reference, and site and mobile Radiographic leads were charged with increasing awareness amongst staff groups of the limits of their scopes of practice and to clearly delineate roles and responsibilities.

**Table 5.** Summary of example alerts arising from the incident management programme

**Alert**	**Event**	**Root cause**	**Outcome**
1.	A near-miss occurred when the safety checking processes failed and the patient, who had a cardiac pacemaker *in situ*, was taken into the scan room. This was detected by the MR Supervisor at the last minute who removed the patient before they were placed within the magnet.	Poor In-Team Communications causing confusion around team roles due to too many staff involved in the care of the patient.	Alert sent to remind staff that whilst trainee and support staff can assist patients with form completion and provide a preliminary assessment of safety, ultimate accountability falls to the MRI Radiographers working in and supervising that Controlled Access Area at the time. Trainees and support staff should be aware of the limits of their scope of practice and delineation of roles and responsibilities should be clearly outlined locally, along with the overall Operational Policies.Introduction of an adapted “Have you PAUSED and checked” based on the Society of Radiographer’s campaign to act as aide memoire for staff when dealing with patients to ensure all suitable checked are carried out throughout the examination.
2.	A patient was unknowingly scanned with an unknown aneurysm clip *in situ* before being recognised on scans and removed from the scanner.	Lack of recall despite multiple protective screening barriers. The GP did not highlight the patient history nor did the patient recall any surgery. When the patient did recall surgery on the day she was still unaware of the clip *in situ*. Demonstrating the potential issues when relying on human memory and necessity of screening patients multiple times with different wording.	The safety screening form, whilst not intended to be a comprehensive check list read verbatim, was mid transition to a modified version using more open questions to help elicit more information from the patient and minimise the potential of missing important medical history.An alert to staff was also issued reminding them of the fundamental principle of our approach to patient safety that when there is any doubt, we must investigate further in order to obtain evidence and not purely take the patients word. We fully understand the medical terminology and the reasons for having to ask specific questions. Patients however cannot be assumed to have a similar level of knowledge and the information they believe can be incomplete or incorrect.
3.	Accompanying nurse from the wards attempted to bring an unchecked oxygen cylinder into the magnet room despite being briefed beforehand in relation to safety around the MRI and specifically the oxygen cylinder. After being stopped by radiographers from taking cylinder any closer to the machine and potentially causing serious harm to both patient and equipment the nurse was unapologetic and did not seem to understand the gravity of the situation despite all the prior warnings.	Lack of awareness and appreciation for the potential severity by the accompanying nurse despite being given advice and warning by MR staff.	A rapid alert was issued to raise awareness of this potentially serious event, in particular raising consideration of safe management of transient items within the MRI controlled access areas. Labelling is used for departmental equipment but is less well considered when items are brought with patients, such as wheel chairs and oxygen cylinders. Suggestion of having labelling available for hanging on equipment was made, or potentially tethering equipment so it can’t be moved. The exclusion of non-MRI staff from waiting within the Controlled Access area was also made.
4.	Following refusal to scan a patient due to the presence of an ICD, the patient’s wife attended the department to say her husband has been scanned a few months ago with it *in situ*. On review of the patient’s paperwork this had not been documented and should not have occurred as there was no pathway in place locally to manage this and ensure the device was safe etc.	Lack of clarity over roles within the screening process and incomplete documentation. Importance of active discussion with patients around their medical history and style of questioning to ensure reliable recall of history.Importance of clear documentation and record keeping if any responses are corrected or incorrect on the screening form.Reiterate the role and responsibilities of Rad Assistants as MR Environment Authorised Personnel and radiographers as Supervisor MR Authorised Personnel.	A rapid alert was issued as a reminder on staff roles and responsibilities within departments and the MRI Screening process. Similar to the first event 18 months prior, this was around suitable use of support staff in pre-screening and patient prep, but ultimate responsibility resting with the MR Supervising radiographer to check details and speak with the patient before entering the magnet room.
5.	A spate of heating-related issues across the organisation within a 3-month period, in particular one associated with a patient feeling warm whilst wearing a metallic flecked jumped for a scan which should have been removed and potentially resulted in a small skin burn at a potential contact site with the jumper material. Another being a conductive loop where the patient moved their hands together mid scan causing a burn.	Appropriate patient preparation and positioning is needed to ensure the risk of any burn occurring is mitigated. The patient jumper should have been removed and as much as possible patients need to be reminded to not link hands whilst during the examination.	Due to the small collection of similar themed events and complaints a rapid alert was issued to remind staff to be vigilant when prepping and positioning patients. Heating and burns will also provide the content for the next communication programme during MRI Safety Week 2018 so that we can share learning and avoid these preventable events from occurring.

ICD, implantable cardioverter defibrillator.

Alert 3 emphasises the gate keeping role of the MR staff. All staff groups were reminded of the operation of the Controlled Access Area and the existing guidelines and procedures. The incident highlighted the key supervisory role of the trained “Authorised Person” members of staff working within the Controlled Access Area. The alert allowed staff to be reminded of the potential lack of awareness of visiting staff groups to MRI and the fact that they often have no perspective of the potential dangers and are often judged as considering the MRI staff as being overzealous in their supervisory MRI safety roles. This incident is an important example of a near miss for a potentially very serious incident. It could have been possible for the incident in question to have been considered by the staff to not be a reportable incident as the radiographer had successfully challenged the nurse and avoided an accident. Staff are constantly reminded of the importance of reporting near misses to allow the safety framework to be reviewed and strengthened as required. During early 2016, there was an internal communications drive to all staff within the organisation to remind them of the importance of reporting near misses in order to increase the visibility of systematic weakness’ within the safety framework.

Alert 4 demonstrates further potential problems resulting from a lack of clarity over roles within the screening process, along with the pitfalls of incomplete documentation regarding the patient’s previous clinical history. The rapid alert which was sent out served to highlight these issues to staff and encourage effective communication between all staff members involved in the safety screening process. An appropriate and flexible style of questioning is essential to ensure reliable recall of history when talking to patients. Closed style questions or technical descriptions can prevent patients from sharing all the necessary information required from the screening interview. Furthermore, the importance of clear documentation and record keeping if any responses are corrected or incorrect on the screening form is important for patients’ possible future MRI referrals.

Alert 5 highlights the need for staff vigilance in relation to patient clothing and patient positioning during the scan (including potential changes to the patient positions of arms and legs, *e.g.* during the scan). Awareness of appropriately checking a patient’s clothing is important, particularly given the increase in metallic based attachments to clothing and metallic fibres within sports/technical garments. In general, patients should remove all excessive layers of clothing to assist assessment and minimise risks. Arguably, the ideal situation would be for all patients to change into specialised “pocket-less scrubs” to reduce risks associated with clothing and also to minimise potential skin to skin contact which can result in conduction loop heating, but this is an acknowledged operational challenge where a pragmatic approach has to be taken.

## Discussion

### Key themes and actions taken

Whilst there is extensive literature around MRI safety incidents and documented cases of adverse events, there is a lack of published data relating to incident rates and trends. Mansouri^8^ demonstrated 5% of their hospital incident reports to be MRI related with a report rate of 0.35% per patient. The total proportion is far lower than that in this review due to the wider nature of workload performed in the hospital setting; however, the incidence report rate from this review of 0.33% specific to MRI is in line with that found.

Of particular interest was the percentage of reports logged between static locations and the mobile services, with almost three quarters being from static units. This is likely explained by differences in information technology and connectivity issues. Logging events is slower and more time consuming on mobile units. This discourages mobile staff from reporting events as they occur. The type of work performed in static units may also be more complex, with more contrast-enhanced studies, a greater patient workload and potentially patients with more challenging biomedical devices being referred. Close management within static units and visibility of management and senior support may also encourage more active reporting of events. Also, the source of the remaining 1% of the MRI reports were from events either notified via head office or the patient referral centre. The referral centre provides clinical triage of referrals with any unexpected devices or safety issues ideally being logged for assessment and investigation but are knowingly under reported.

Sadigh^9^ looked at what they described as unexpected events in MRI with an occurrence of 16.7% (10.4% excluding contrast agent-related events) but their classification of an event was much wider than specifically MRI safety and contrast-related issues. They specifically reported on cases of unanticipated foreign bodies which accounted for 2.96% of cases which is also in line with the 3% reported by the MHRA^11^ for foreign metal objects. These are much lower than our findings which reported 10% of cases to be related to unexpected implants or foreign bodies seen on the MRI images.

We found only 15.5% of our logged MRI incident reports were specifically MR safety related, with the remaining majority associated with booking errors, health & safety, and abuse & harassment. The specific MRI safety-related incidence rate noted in this review was 0.05% (1 in every 1987 patients) which is likely to be a significant under reporting.^1,3^ However, even if it were to be doubled or tripled to account for this, it is still far lower than hospital incident rates where incidents occur in around 10% of patients.^8^ This therefore suggests that in general, MR-related safety within the organisation is well managed.

From a review of the subcategory breakdown, the biggest issue was related to contraindicated referrals (216/674 = 32%), particularly from General Practitioners, concerning pacemakers. This is likely to be as a consequence of confusion over MR conditional devices that not all sites currently scan. A review by Dewey^12^ showed the referral frequency of contraindicated patients to be 0.41%, with pacemakers being one of the main reasons for deferred scans (0.08%) along with shrapnel. Sadigh et al (2017) also noted a 0.43% incidence of pacemaker referrals (but only making up 0.07% of reports). Both reports showed a greater overall incidence rate per patient than the 0.016% incidence noted in this review, but making up less of their safety events logged overall. There are fewer absolute contraindications for MRI nowadays than there once was, with many now being considered relative and dependent upon their conditionality for safe scanning. These events which are suspected under reported still represent one-third of event reports seen and demonstrate that education of referrers is still needed along with clear information given to patients for their own awareness.^12^

**Figure 3.**
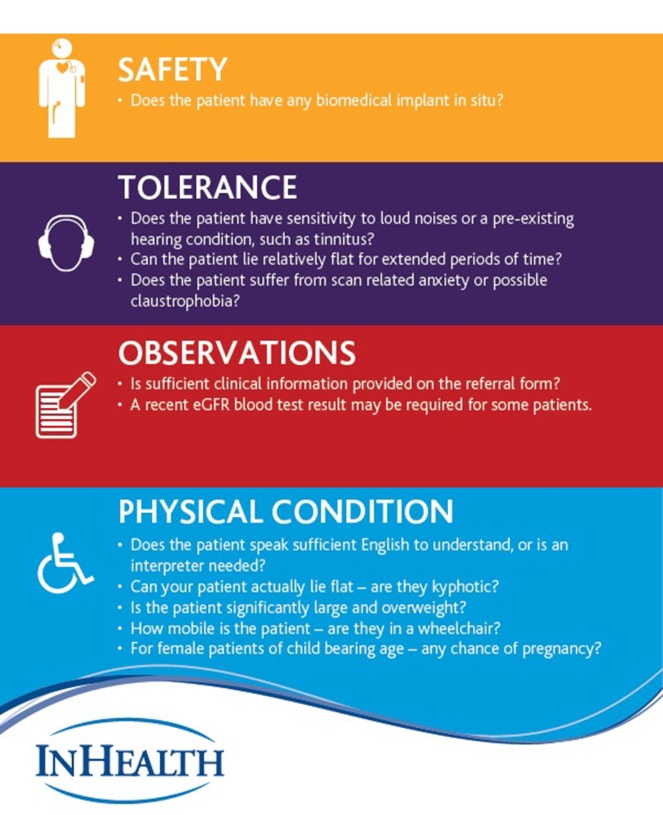
Referral guidance document developed to support education of referring clinicians, based on the acronym STOP.

A peak in contraindicated referrals was noted in the first half of 2017 which dropped again in the second half in part due to increased education of referrers. For MRI Safety Week 2017 (a week chosen to raise awareness of MRI safety that also coincides with the anniversary of Michael Colombini's death in 2001), a referral guidance document was developed to support education of referring clinicians (Figure 3), based on the acronym STOP; Safety (does the patient have any biomedical implants *in situ*), Tolerance (are they aware of the acoustic noise or are they claustrophobic?), Observations (is sufficient clinical information provided and an eGFR where needed?), and Physical Condition (can they lie flat or do they have communication difficulties?). This was shared with all departments across the organisation and its use is encouraged in response to contra indicated referrals and in local induction for junior doctors.

To further support the findings regarding devices, local pathways for the safe management of MR Conditional cardiac devices has been established at some sites, where support is available, across the organisation. Those sites with high deferral rates are encouraged to discuss appropriate pathways with local cardiology departments to explore if these could be managed safely and effectively. At present, the main areas where this is limiting is in community based locations outside of the hospital setting, and those services delivered from mobile units. As developments in devices continue to improve and checking processes by manufactures become more simplified, it is envisaged that in time management of these devices for safe scanning will become more common place within the wider MRI community.

Categories of “unable to confirm safety” and “unexpected implant/foreign body” (21.5% collectively), suggest issues associated with screening processes and interaction with patients. These failures in communication around devices *in situ* is the next most significant issue, highlighting that accurate recall of medical history from referrers and patients is fundamental. Referrers do not always recognise or understand the importance of specific checks, and patients do not always understand the medical terminology used and are frequently unable to recall past interventions.^1^ Patients should ideally be questioned twice along their referral pathway with a final check by the MRI Radiographers.^2^ The key message from these findings seems to be related to the importance of effective patient engagement and safety screening in order to be able to obtain a reliable and adequate medical history to be able to assure patient safety.

No cases of harm have been recorded for foreign bodies first seen at MRI, even for those considered to be intraocular in nature (38% of the reported unexpected foreign body cases). Unfortunately, no screening process is perfect and so it is likely it will fail on occasion. The reason for failure is most likely due to human error. The lack of reported harm may also suggest that such instances are likely to occur unknowingly.^13^ A review by Eshed^14^ on retained metal fragments from combat and terrorist attacks showed that over a 10-year period there was no resultant harm to any patients undergoing an MRI. Precautions over size and location still need to be applied but this does demonstrate that the probability of harm is low.

Screening for intraocular foreign bodies (IOFB) has always been a difficult issue and generally there is no standardised approach within the UK. Following a study by Seidenwurm,^15^ the requirement for additional imaging before undergoing an MRI examination has reduced and become more balanced in approach, relying first on more effective questioning and obtaining a history of the injury to better assess risk before deciding to proceed with MRI or the need for pre-screening imaging. The general approach used is based on this study, and unless penetrating in nature and untreated, the risk of retained fragments is considered to be low and therefore it is safe to proceed with the examination. Although that’s not to say that verbal screening will pick up all patients at higher risk of IOFB.^16^ As outlined by Bailey & Robinson,^17^ human factors can play a part, such as lack of recall, communication barriers, or withholding information. Despite this, there have been few cases of harm recorded as a result of undetected IOFB; a potential case of cataract formation resulting from fragment movement in 2001 which was missed on pre-screening radiographs,^18^ and the most recent report of hyphema in 2015 following confirmed removal of IOFB and subsequent MRIs.^13^ Other cases reported have shown that despite evidence of retained fragments detected on MRI, no harm has resulted.^19^ Potential for harm, particularly from occupational injury, is further reduced these days as a result of tighter Health and Safety requirements around the use of Personal Protection Equipment, such as goggles^20^ when performing tasks involving metal-working.

**Figure 4.**
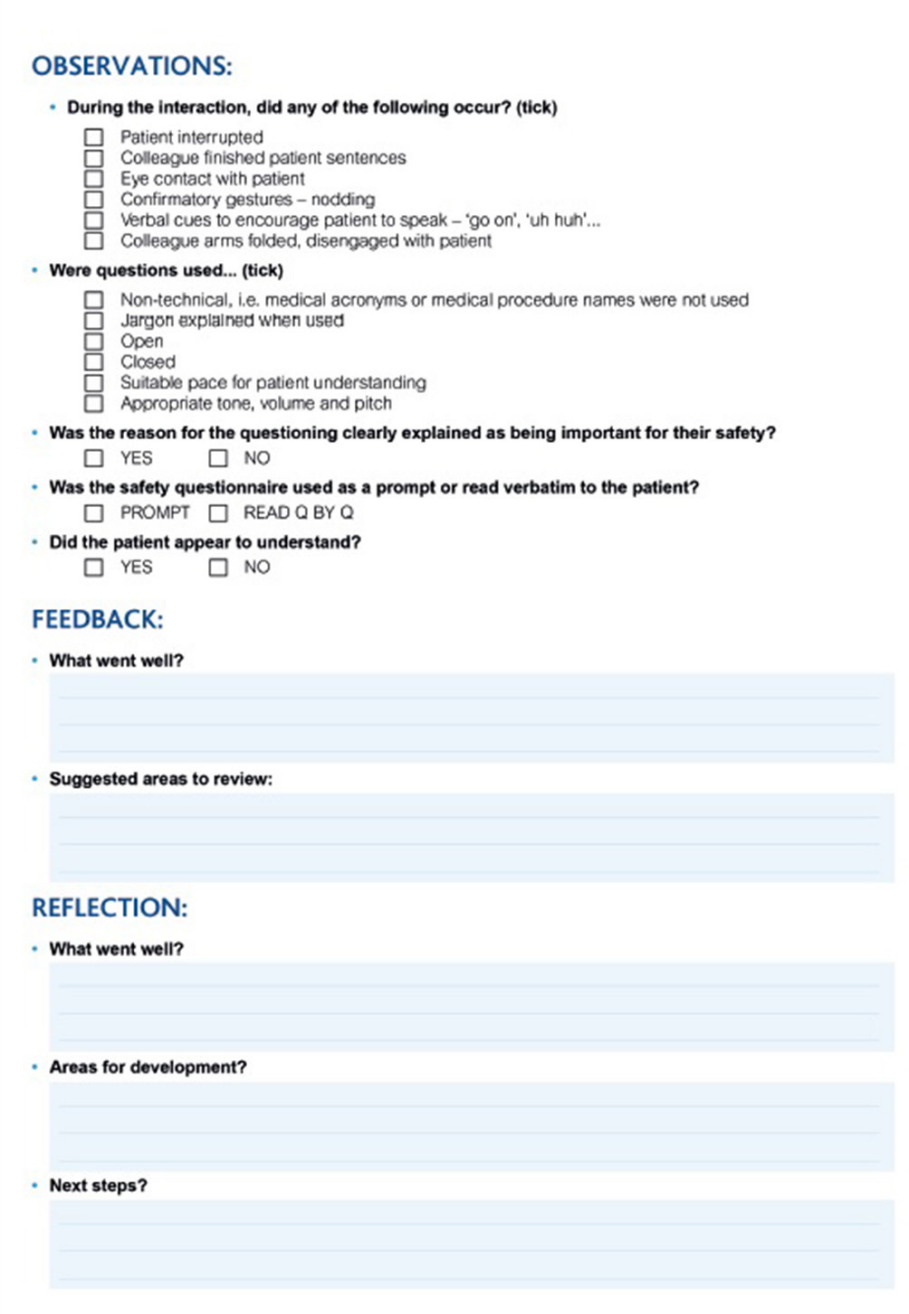
The supporting checklist and form that staff were encouraged to use as part of the peer review process established to explore communication skills during the patient safety screening process.

To support communication skills and effective screening techniques, staff were also asked during MRI Safety Week 2017 to participate in a peer review of each other’s safety screening approach (highlighted in “Discussion” around rapid Alert 4). Figure 4 demonstrates the supporting checklist and form that staff were encouraged to use as part of the peer review process. Completed paperwork remained anonymous in terms of staff names but they were collated and reviewed at a local level and at an organisational level for those forms shared with head office. Any screening interaction with a patient is a two-way process built around ensuring they understand the questions being asked and their importance, as well as being listened to with their replies. It comprises of verbal and non-verbal cues which build up a sense of "intuition" around how reliable a patient’s response may be. Radiographers need to confidently assess patient communication as well as being able to communicate appropriately themselves. The skill is tailoring the communication approach appropriately to each patient interviewed. The aim of the observation was to provide opportunity to review and reflect on one’s practice and communication with patients. It was important any observations made were objective and non-judgmental; there can be several ways of successfully communicating with any particular patient. This task was not designed to be critical of colleagues but about personal improvement and development. Reflection and peer review have the potential to provide improvements in clinical practice, be invaluable for continuing professional development and support patient safety and quality, along with opportunity to update knowledge and skills.^21^ All of which support delivery and evidence of competent clinical practice in line with the Health and Care Professions Council requirements.

Communication skills within MRI are increasingly being recognised as essential to so many general aspects of the procedure; not only in ensuring safety for patients and staff. Establishing rapport with patients to alleviate any anxiety and support compliance is key to a successful examination and to the experience for the patient. This connection should continue throughout an examination.^2^ It is another area where staff support has been focused internally both through Human Factors training to support self-awareness and safety, as well as through development of patient experience resources to support scan anxiety.

Whilst the peer-review tool was not necessarily ideal, from review of some of the peer feedback and reflections shared by staff, it did appear to prove useful to some. Common themes highlighted in the observations were a tendency to direct closed questions or pose them in a negatively suggestive way, *i.e*. “You don’t have any pacemaker, do you?”. It was also felt by some that the experience was useful to provide focus in normally busy days to reflect on how they actually ask patient’s questions for safety in practice. It seemed to promote the desired outcome and prompted discussion amongst staff to share practices and improve.

Many of the recorded events related to an inability to confirm patient safety were connected to lack of awareness misinterpretation of the organisational safety policy, which does provide some generic overarching caveats on specific devices and implants for streamlining efficiency whilst still ensuring safe scanning. Other recorded incidents were related to considerations over management of examinations within six weeks post operation. This 6-week criteria has historically been considered the safe period after which most inserted devices or implants could be scanned as this related to the general time it takes tissue fibrosis to develop and support retention of any items.^22^ However, increasingly, device testing and experience are showing that in many circumstances there is no need to delay scans and that risks from potential hazards associated with the imaging process are negligible. As a result, internal guidance was updated with the aim of supporting a safe but pragmatic approach to patient management without causing unnecessary delays in care.

The events associated with projectile incidents and burns were low, 4.74 and 1.48% respectively. The only reference for comparison is data from the MHRA^11^ which shows 41% of reports being related to burns and 17% related to projectiles. It is acknowledged that these are reported at a national level due to the harm caused to patients or severity of projectile incidents occurring. Most projectile events or near misses are likely not reported,^5^ resulting in less serious events being unreported at any level.^6^ Across the organisation, we have had three actual burns noted as a result of the scan, whereas more actual or near miss projectile events occur which are predominantly of a minor nature (*e.g.* Table 2). Many burns can be prevented and managed through correct positioning within the scanner.^5^ However, management of projectiles can be more problematic, especially on mobile units with increasing operational pressures and because of the confines of the physical space. Ideally, to eliminate many of the projectile events seen, all patients would be appropriately changed to ensure attached items were removed and pockets emptied.^10^ This would also minimise the potential risks of related burns or artefacts on images.^2^ In practical terms, however, this is not always possible due to the physical constraints of some departments, such as when working on mobile units or due to operational time constraints. Previous discussions around the rapid Alert 5 have highlighted these issues.

Another consideration, advocated by the ACR^23^ and a potential suggestion by the MHRA,^10^ is the use of Ferromagnetic Metal Detectors. A study by Orchard^3^ showed the use of these to be 100% sensitive and 98% specific when used in clinical practice, with some items being detected that may have been missed using standard screening methods. The difficulty with practically implementing these screening devices into the clinical department is often due to department design limitations, such as on a mobile unit, and other considerations regarding the need for both patient changing and metal free staff uniforms.^2^ MRI safe equipment may also contain small ferromagnetic components which do not compromise their safety but would trigger an alarm. In these circumstances, experience has shown that unnecessary alarming causes “alarm fatigue”^3^ with a consequential reduction in integrity of the systems use. Another limitation is that whilst useful for prevention of projectile events, they still leave the risk of non-ferrous items which could cause heating-related issues.^24^ Therefore, whilst a useful adjunct and tool in the screening process at some centres, their use needs to be made clear so that staff use them appropriately^24^ as they are not a replacement for current screening practices and suitable patient interaction to ascertain safety.^3^

Suitable equipment labelling is also recommended by the MHRA^10^ in line with the ASTM standard. As with notable Case 3, another event highlighting the importance of clear labelling involved a non-MR-Safe wheelchair being inadvertently taken into the entrance of the magnet room before being stopped by fellow staff. On investigation, the staff member was aware of the safety requirements but due to the chair being almost identical to their MRI Safe one and the list running late which added additional stress and distraction, the standard processes failed. This example goes beyond simple ASTM labels as they were in use within the department but not looked for due to other surrounding factors. Potentially, other adjuncts which could help may be making the types of wheelchairs more obviously different, or using coloured tape around handles to make its nature more evident.

The authors question the application of the general definition of equipment labelling to both biomedical implants or devices and inanimate equipment items or objects in the MR Controlled Access Area. Whilst metallic components of an implant or device should be clearly considered MR Conditional to ensure due care and attention is given in terms of managing risks to patients, the application of this rule, for *.*to a non-ferromagnetic wheel chair that is designed to be used fully within the MR Environment, potentially confuses the care required when using formally MR Conditional equipment items such as patient monitors or anaesthetic equipment. The labelling of equipment within the MR Controlled Access Area could benefit from a simpler definition where non-ferromagnetic items can be assessed and labelled as MR Safe for the specific installed MRI system. The important use of MR Conditional labels would then remain significant to ensure staff exercise the appropriate care and restrictions when using that particular equipment.

Issues relating to acoustic noise were also low at 0.89% of reported MR Safety events, far lower than the 3% reported by the MHRA.^11^ Those logged were predominantly complaints from patients following their scan and not concerning adverse short or long-term hearing loss. The concerns were mainly around lack of explanation and poorly fitting ear protection. Correctly fitting protection is important as studies have shown that this prevents permanent hearing loss, with small changes noted immediately after scan but returning to normal over preceding weeks.^25^ Appropriate training of staff in the correct use of ear protection is paramount,^10^ as patients do not necessarily know how to apply them correctly and so require clear instruction to ensure maximum effectiveness.

Staff training is supported through the development of in-house electronic learning materials. Table 6 summarises the three modules aimed at addressing the different levels of training required in line with the MHRA Guidance.^10^ The training is added to staff’s mandatory training records and should be carried out annually. Completion also assists in the provision of documentary evidence that staff have received an appropriate level of safety training (in particular ear protection and RF coil safety). The electronic learning materials can be supported by local teaching sessions. Implementation of these organisational-wide educational support tools helps to ensure a specified level of knowledge around MRI Safety for all staff groups. Furthermore, in addition to contributing to the protection of both staff and patients, there is an important role in further promoting a culture of safety within the MR facilities.^6^

**Table 6.** Educational module structure used to support awareness and knowledge relating to MR safety

	**Audience**	**Objectives**
**Module 1**	ALL categories of staff working within MRI—administrative staff, drivers, clinical assistants, porters and radiographers. Including MR Safety Video.	Awareness of the location of the MR Environment and its hazards.Safety aspects relating to the static magnetic field—projectile effect, interactions on implants and equipment, and personal effects such as credit cards and watches.Understanding of the significance of the MR Controlled Access Area and MR Environment, and be able to differentiate them.
**Module 2a**	All Clinical Staff directly involved with patients attending for MRI—MR Radiographers/Practitioners and Assistants. Inc. Earplugs Instruction Video.	Understanding of safety aspects related to radiofrequency and time-varying gradients.Awareness of managing these risks, including correct patient preparation and positioning.Instruction in correct selection, fitting and use of ear protection.
**Module 2b**	All MRI Radiographers/Practitioners operating scanners and working within MRI	Understanding of emergency procedures arising from causes other than equipment failure.Understand local regulations and procedures in connection with the MR diagnostic equipment and its location.Understand the consequences and effects of quenching of superconducting magnets.Awareness of the recommendations over scanning modes and exposure to MR.

The nature of the recorded events related to GBCAs were mostly mild with none considered as being severe. The ACR guidelines^26^ state that reaction severity is very much subjective and not simple to stratify into classes. The guidance suggests; mild reactions would be *self-limited without evidence of progression*, moderate are *more pronounced and commonly require medical management*, and severe are *life threatening*. Whilst there was no adverse reaction rate to compare with data, the percentage of contrast reactions reported accounted for 18.4% with a rate overall per patient of 0.0093%. This is similar to what Mansouri^8^ noted in their study making up 19.1% of reports but their overall rate higher at 0.068%. Comparing also with the study by Sadigh,^9^ they showed a much lower overall percentage of reports being reactions (2.32%) but with an overall incidence rate across all exams conducted to be higher at 0.4%. The usefulness of this data is limited by the fact that it relates to total examinations performed and not specifically those receiving contrast. Variation in severity of reactions is expected due to variability in the types of contrast agents and the preparation used (chelates and additives used), with a review by Behzadi^27^ showing higher reactions occurred with ionicity, protein binding and macrocyclic structures. The papers reviewed in their analysis showed 81% of immediate reactions noted were mild, 13% moderate, and 6% severe. Our data are comparable with 89.5% considered insignificant/mild, 10.5% moderate, and zero severe cases—this is based on predominate use of Dotarem (gadoterate), followed by Gadovist (gadobutrol), and some use of Prohance (gadoteridol) and Primovist (gadoxetate). The main cautionary note to consider when looking at severity ratings is disparity amongst staff as to what should be considered mild or moderate, whilst most staff would agree severe reactions are any that result in anaphylaxis, patient harm, or admission to hospital as a result.

Similarly, for extravasations, these accounted for 1% of the incidents with a rate of 0.0044%, whilst Sadigh^9^ report 0.58% of incidents to be extravasations and a rate of 0.1%. Interestingly, Mansouri^8^ found their incidence rate over all examinations to be low at 0.044% but extravasations made up 12% of their reported incidents. Although making comparisons with reported rates is difficult due to the variation in what constitutes an MRI safety event. The main reason on both counts might be that staff more readily report medicines-related incidents when they occur because they see this as being more important and necessary to log as opposed to logging events relating to MRI safety. Management of both adverse reactions, through logging via the MHRA Yellow card system, and of extravasation, through effective clinical management, are well-recorded and followed by staff. A gradual increase in extravasations may also be seen as a result of increased workload requiring use of a power injector, and encouragement of their use as best practice^10^ and also supporting the requirements of the Control of Electromagnetic Field at Work Regulations requirements.^28^

For both adverse reactions and extravasations involving GBCAs, internal discussion has been had around providing staff with standardised report templates to ensure required information is included in any of these reports. It would also support clearer classification in terms of reaction severity which would improve quality of data extracted and comparison.

Whilst the review has proved invaluable for the assessment of MRI safety within the organisation, it is acknowledged that the chosen subcategories are far from perfect and do present limitations, as discussed, around how some events fit into any one category. The first two categories for projectiles and equipment labelling could arguably be amalgamated into one, however, they were separated in order to help highlight any issues around lack of labelled ancillary equipment and staff awareness of these, but these would then lead to potential projectile issues. Faulty coils and oxygen monitors could also be either combined under one faulty equipment category and removed considering there were no reports logged and they are ultimately managed through a fault reporting system instead. From review of the “other MRI-related” category, it could also be suggested that one for pregnancy may be of use for wider learning of related issues.

## Conclusions

This review has presented findings relating to MR safety incidents covering approximately 1.3 million patients scanned, with a total number of 4343 MRI-related safety event reports submitted. 72% of incidents were related to static MR sites and 27% were related to mobile MR units (with 1% relating to head office or call centre reports). The reporting rate overall for MRI incidents was 0.33% (1 in 308 patients). 15.5% of the logged MRI incident reports were specifically MRI safety related, with the remaining majority being connected to booking errors, health & safety, and abuse & harassment. The specific MRI safety-related incidence rate noted in this review was 0.05% (1 every 1987 patients).

Analysis and visibility of MR safety-related incidents has allowed possible weaknesses in process to be identified. Where processes have been highlighted as requiring changes initiatives were usefully instigated to strengthen safety procedures and the overall safety culture within the organisation. Structured staff training has been successfully used to develop staff awareness and the necessary skills and knowledge to effectively manage MR safety. Initiatives arising from the experiences of this review were also shared across the wider MR community in the UK via a variety of routes, including conference presentations and social media.

Our experiences of the benefits of analysing safety incidents and subsequent information sharing within the organisation suggest that wider sharing of MRI safety-related events, as well as continued data on contrast agent reactions and wider incidents, will provide benefits for the MR community in general. This is entirely in agreement with previous reported findings that the MRI community can learn from one another and establish good practices to help mitigate flaws in processes and human fallibility.^1^

The most prevalent events seen were around contraindicated referrals, followed by adverse contrast reactions and failings in the screening process. Therefore, continued education, of referrers and staff, along with development of effective communication skills are the key lessons to be learned.

Unlike radiation incidents and drug reactions, there is no UK mandate to report or monitor local MRI safety-related events to either the MHRA or Care Quality Commission, therefore report rates are *ad hoc* and significantly less than what is likely to be occurring. *“Defining an industry wide classification scheme for incident reports in Diagnostic Imaging would allow for better inter institutional comparison and development of national performance benchmarks* ^8^

11 years on from the analysis conducted by De Wilde,^5^ similar conclusions can be drawn, and occurrence of incidents seen in MRI across the organisation are low, suggesting on the whole that safe practices are followed and safety is well managed. Overall, adverse events are a result of human error in one form or another.

Continual review of internal safety data for MRI helps the organisation manage safety and intervene as appropriate. Feedback on occurring safety events is an important aspect in closing this loop of learning which is mainly managed through rapid alerts, although more regular sharing of data would be beneficial to all staff. We believe that if more departments adopted this approach there would be a greater level of sharing of information and best practice, with obvious clear benefits to MR clinical services, staff and of course patients.

## References

[1] Watson RE, Watson RE Lessons learned from MRI safety events. Curr Radiol Rep 2015; 3: 37. doi: 10.1007/s40134-015-0122-z

[2] Weidman EK, Dean KE, Rivera W, Loftus ML, Stokes TW, Min RJ MRI safety: a report of current practice and advancements in patient preparation and screening. Clin Imaging 2015; 39: 935–7. doi: 10.1016/j.clinimag.2015.09.002 26422769

[3] Orchard LJ Implementation of a ferromagnetic detection system in a clinical MRI setting. Radiography 2015; 21: 248–53. doi: 10.1016/j.radi.2014.12.007

[4] Kraff O, Ladd ME MR Safety Update 2015: Where do the risks come from? Curr Radiol Rep 2016; 4: 34. doi: 10.1007/s40134-016-0163-y

[5] DEWILDE J, Grainger D, Price D, Renaud C Magnetic resonance imaging safety issues including an analysis of recorded incidents within the UK. Prog Nucl Magn Reson Spectrosc 2007; 51: 37–48. doi: 10.1016/j.pnmrs.2007.01.003

[6] Crisp S, Dawdy K, Crisp S, Dawdy K Building a magnetic resonance imaging safety culture from the ground up. J Med Imaging Radiat Sci 2018; 49: 18–22. doi: 10.1016/j.jmir.2017.10.005 30479282

[7] Schultz SR, Watson RE, Prescott SL, Krecke KN, Aakre KT, Islam MN, et al Patient safety event reporting in a large radiology department. AJR Am J Roentgenol 2011; 197: 684–8. doi: 10.2214/AJR.11.6718 21862812

[8] Mansouri M, Aran S, Harvey HB, Shaqdan KW, Abujudeh HH Rates of safety incident reporting in MRI in a large academic medical center. J Magn Reson Imaging 2016; 43: 998–1007. doi: 10.1002/jmri.25055 26483127

[9] Sadigh G, Applegate KE, Saindane AM Prevalence of unanticipated events associated with MRI examinations: a benchmark for MRI quality, safety, and patient experience. J Am Coll Radiol 2017; 14: 765–72. doi: 10.1016/j.jacr.2017.01.043 28356198

[10] MHRA Safety Guidelines for Magnetic Resonance Imaging Equipment in Clinical Use; 2015.

[11] MHRA Update Session delivered at IPEM conference; 2017.

[12] Dewey M, Schink T, Dewey CF Frequency of referral of patients with safety-related contraindications to magnetic resonance imaging. Eur J Radiol 2007; 63: 124–7. doi: 10.1016/j.ejrad.2007.01.025 17383136

[13] Lawrence DA, Lipman AT, Gupta SK, Nacey NC Undetected intraocular metallic foreign body causing hyphema in a patient undergoing MRI: a rare occurrence demonstrating the limitations of pre-MRI safety screening. Magn Reson Imaging 2015; 33: 358–61. doi: 10.1016/j.mri.2014.12.009 25523608

[14] Eshed I, Kushnir T, Shabshin N, Konen E Is magnetic resonance imaging safe for patients with retained metal fragments from combat and terrorist attacks? Acta Radiol 2010; 51: 170–4. doi: 10.3109/02841850903376298 19912071

[15] Seidenwurm DJ, McDonnell CH, Raghavan N, Breslau J Cost utility analysis of radiographic screening for an orbital foreign body before MR imaging. AJNR Am J Neuroradiol 2000; 21: 426–33. 10696035 PMC7975348

[16] Jessop S, Hart G, Santiago AR, Samara A, Markali B, Cottier Y et al Review Article – X Radiation dose implications in screening patients with ferromagnetic IOFBs prior to MRI: a literary review : OPTIMAX 2014 – radiation dose and image quality optimisation in medical imaging. Lisbon; 2014 53–9.

[17] Bailey W, Robinson L Screening for intra-orbital metallic foreign bodies prior to MRI: Review of the evidence. Radiography 2007; 13: 72–80. doi: 10.1016/j.radi.2005.09.006 33383605

[18] Vote BJ, Simpson AJ X-ray turns a blind eye to ferrous metal. Clin Exp Ophthalmol 2001; 29: 262–4. doi: 10.1046/j.1442-9071.2001.00420.x 11545429

[19] Zhang Y, Cheng J, Bai J, Ren C, Zhang Y, Gao X, et al Tiny ferromagnetic intraocular foreign bodies detected by magnetic resonance imaging: a report of two cases. J Magn Reson Imaging 2009; 29: 704–7. doi: 10.1002/jmri.21637 19243045

[20] Health & Safety Executive Personal Protective Equipment at Work. 3rd Edition HSE: The British Institute of Radiology.; 2015.

[21] Jones DD, Jones DD Peer observation: a tool for continuing professional development. Int J Ther Rehabil 2007; 14: 11.

[22] IMRSER Guidelines for the Management of the Post-Operative Patient Referred for a Magnetic Resonance Procedure. 2018 Available from: www.IMRSER.org.

[23] Expert Panel on MR Safety, Kanal E, Barkovich AJ, Bell C, Borgstede JP, Bradley WG, et al ACR guidance document on MR safe practices: 2013. J Magn Reson Imaging 2013; 37: 501–30. doi: 10.1002/jmri.24011 23345200

[24] Keene MN, Watson RE, Keene MN, Watson RE Ferromagnetic detectors for MRI safety: toy or tool? Curr Radiol Rep 2016; 4: 20. doi: 10.1007/s40134-016-0146-z

[25] Salvi R, Sheppard A Is noise in the MR imager a significant risk factor for hearing loss? Radiology 2018; 286: 609–10. doi: 10.1148/radiol.2017172221 29356640

[26] American College of Radiologists ACR Manual on Contrast Media – Version 10.3: The British Institute of Radiology.; 2017.

[27] Behzadi AH, Zhao Y, Farooq Z, Prince MR Immediate allergic reactions to gadolinium-based contrast agents: a systematic review and meta-analysis. Radiology 2018; 286: 471–82. doi: 10.1148/radiol.2017162740 28846495

[28] Health & Safety Executive The Control of Electromagnetic Fields at Work Regulations, 2016, No 588. HSE: The British Institute of Radiology.; 2016.

